# Establishment of Diagnostic Protocols for COVID-19 Patients

**DOI:** 10.12669/pjms.36.7.2957

**Published:** 2020

**Authors:** Sana Abbas, Aisha Rafique, Beenish Abbas, Madeeha Sattar

**Affiliations:** 1 Dr. Sana Abbas, MCPS. National University of Medical Sciences (NUMS), Rawalpindi, Pakistan; 2 Dr. Aisha Rafique, MBBS. National University of Medical Sciences (NUMS), Rawalpindi, Pakistan; 3 Dr. Beenish Abbas, FCPS. Foundation University, Rawalpindi, Pakistan; 4 Dr. Madeeha Sattar, BDS Foundation University, Rawalpindi, Pakistan

**Keywords:** Coronavirus, Coagulation Profile, C-reactive Protein, Fever, Ferritin, Leukocyte

## Abstract

**Objective:**

To evaluate the oscillation of laboratory parameters among indoor patients infected with COVID-19.

**Methods:**

This cross-sectional analytical study was conducted at Tertiary Care Institute, Rawalpindi from 01 March 2020 to 20 May 2020. Three hundred and ninety-two patients with mild to moderate illness, PCR positive for COVID 19 were included. Prevalence of typical symptoms of coronavirus disease cough, fever, sore throat and shortness of breath was recorded.PCR was repeated after seven days of admission, if declared negative, another executed on consecutive day. Discharge Criteria was two consecutive negative PCR.

**Results:**

A total of 392 patients enrolled in the study with age range 9-45 and mean 33.22±7.98 years. A total of 8 (2%) patients were females and 384(98%) males. 296(75.5%) did not have a cough whereas 96(24.5%) presented with the trait. 296 (75.5%) patients did not have associated fever whereas 96(24.5%) manifested with fever. Chest x-ray had a bilateral patch in 96 (24.5%) patients only. Ferritin was raised in 96 (24.5%) patients however were recorded within normal limits in 296(75.5%) patients. Coagulation Profile was deranged in 64(16.3%) patients whereas was within range in 328(83.7%) patients. Serum Bilirubin, Serum Alkaline phosphatases, Serum Albumin, Serum Urea, Serum Potassium were essentially in typical tolls in 392(100%) patients. However Serum Alanine Aminotransferase was raised in 32(8.2%), Serum C reactive Protein was elevated in 48(12.2%). An exaggerated values of serum creatinine and serum sodium were noticed in 24(6.1%) and 16(4.1%) respectively.

**Conclusion:**

Routine haematological tests, biochemical tests, serological tests, and radiographic tests are crucial to conclusion, foundation and progression of ailment in COVID-19 contaminated patients.

## INTRODUCTION

Ongoing pandemic is caused by severe acute respiratory syndrome coronavirus 2 (SARS CoV 2). The outbreak was first recognized in Wuhan, China, in December 2019 as pneumonia of unknown aetiology spreading speedily globally.^1^ The World Health Organization acknowledged the outbreak a Public Health Emergency of International Concern on 30 January, and a pandemic on 11 March.^2^ There has been various researchs studies proposing correlation between severity of covid 19 disease and laboratory parameters. The severity of the clinical signs and symptoms of covid infection is directly related to altered levels of C-reactive protein, erythrocyte sedimentation rate, serum lactate dehydrogenase levels, serum albumin levels and D-dimers.^3^ It thus implies fundamental role of haematological and biochemical marker in establishing diagnosis and monitoring disease. It also signifies that Laboratory parameters accurately assess probable outcomes of the disease. However these pieces of information are not uniform in different populations. It will need more evidence based data with evolving times. More significantly, laboratory work up are based on epidemiological studies. These diagnostic investigations derive their influence from effective health care policies and strategies devised at national and international set ups.^4^

Laboratory investigations can be divided into diagnostic markers, prognostic markers and those assessing current or latent infection including immunological markers. The Centers for Diseases Control on Prevention (CDC) from the United States of America (USA) and Hong Kong has developed real-time reverse transcription–polymerase chain reaction (rRT-PCR) assay that can be used to diagnose the virus in respiratory and serum samples from clinical specimens. It received special emergency authorization from FDA when covid 19 started to flood worldwide Various laboratory markers indicate leukocytosis or leukopenia. There may be severe lymphopenia in the early course of disease. Presence of neutrophilia is associated with unfavourable prognosis.^5^

Haematological and chemical laboratory testing plays an important role in diagnosing and managing COVID 19 disease by providing several useful prognostic markers.^6^ The decision to test should be based on clinical and epidemiological factors and related to identification of the possibility of infection. PCR testing of asymptomatic or mildly symptomatic contacts can be considered in the assessment of individuals who have had contact with a COVID-19 case. Screening protocols should be adapted to the local situation.^7^

Rapid collection and testing of appropriate specimens from patients meeting the suspect case definition for COVID-19 is a priority for prompt clinical management and practising outbreak control. It should be guided by a laboratory expert and to be performed in appropriately equipped laboratories by staff well conversant in the relevant technical and safety procedures.^8^ National guidelines on laboratory biosafety should be followed in all circumstances. There is still limited information on the risk posed by COVID-19, but all procedures should be undertaken based on a risk assessment.^9^,^10^

The following study discusses laboratory parameters of various haematological factors which may be within normal limits or raised in COVID 19 positive patients. It also assess individuals who have radiological findings consistent with COVID 19 disease. Severity was positively correlated to the rise of acute phase reactants and radiological findings. These haematological markers were also assessed for monitoring the progression of disease. Generally, patients who had fever and cough at the time of admission had raised inflammatory markers as compared to asymptomatic patients. Similar is the case with the patient with comorbid. Our study aims to aid in the assessment of risk to help in early recognition of the severity of disease and thereby preventing morbidity and mortality.Objective was to evaluate the oscillation of laboratory parameters among indoor patients infected with COVID-19.

## METHODS

This cross-sectional analytical study was conducted at Tertiary Care Institute, Rawalpindi from 01 Mar 2020 to 20 May 2020 approval was taken from the ethical research committee of the Institute (ERC Number – 242/ERC).

The minimum sample size required for this prospective cross-sectional analytical study was 373, calculated by using formula (n = [deff *np(1-p)]/ [(d2/z21-α/2*(n-1)+p*(1-p)]-open epi calculator), with 95% confidence level and 5% margin of error where the hypothesized frequency of raised C – reactive protein related to coronavirus disease was considered to be (58.7%+/-5 ) as reported by Fu et al.^11^ A non-probability consecutive sampling methodology was employed and total (n = 392)participants enrolled in the study.All patients included were PCR positive for COVID 19.

Sample was taken by the throat and nasopharyngeal swabs. Patient was admitted on the first day of positive PCR In a ward completely dedicated to COVID 19 patients. PCR was repeated after 7 days of admission. If it was negative, another PCR was planned on consecutive day. Two consecutive negative PCR were considered fit for discharge. There was adequate spacing between beds of admitted patients. Cohort study with > 44000 patients conducted at China concluded that COVID -19 ailment can range from mild to critical. Our study sample included patients with mild to moderate disease(mild symptoms upto pneumonia constituting 81% of victims as defined by Centers of Disease Control and Prevention).^12^

Patient with comorbid like hypertension, diabetes mellitus, ischemic heart disease, rheumatoid diseases and asthmatic were separately identified and also included in the study. Patients with fever were advised HCQ 400 mg twice a day for two days followed by 200 mg BD for the next 4 days. Alongside oral azithromycin 500 mg OD was given for 5 days. All patients were advised vitamin C, calcium and zinc supplements.

Patients developing severe symptoms of chest tightness, dyspnea and decreasing oxygen saturation of less than 90 per cent were excluded from the study as they were shifted to high dependency unit.

Data was entered and analysed by using data management software IBM SPSS (version 23.0). The descriptive statistics of continuous variables were presented as mean and standard deviation, while categorical data frequencies and percentages were used. Categorical grouped data was analyzed by either Chi-square, Fischer-exact test, and Pearson’s co-relation as applicable. A p-value of ≤0.05 was considered to be statistically significant.

## RESULTS

Total three hundred ninety two patients enrolled in the study with age range 9 - 45 and mean 33.22±7.98 years. A total of 8 (2%) patients were females and 384(98%) males. 296(75.5%) did not have a cough whereas 96(24.5%) presented with fever. Fatigue and myalgias were predominant in 112(28.6%) patients. and were absent in 280(71.4%). Shortness of breath was witnessed in 40(10.2%) patients. Chest tightness was present in 96(24.5%). Comorbid prevalence was hypertension in 40(10.2%), diabetes mellitus in 24(6.1%), and asthma in 8(2.0%) patients. Total Leukocyte Count (TLC) was raised in 96(24.5%) patients. Chest x-ray(CXR) had bilateral patch in 96(24.5%) patients only. Ferritin was raised in 96 (24.5%) patients. Coagulation Profile was deranged and normal in 64(16.3%) and 328(83.7%) patients respectively. Association with predominant clinical manifestations (Fever and Cough) endorsed in (Table-I). Serum Bilirubin, Serum Alkaline phosphatase (ALP), Serum Albumin, Serum Urea, Serum Potassium were essentially in typical tolls in 392 (100%) patients. However Serum Alanine transaminase (ALT) was raised in 32(8.2%), Serum C-reactive protein (CRP) was elevated in 48(12.2%). An exaggerated values of serum creatinine and serum sodium were noticed in 24(6.1%) and 16(4.1%) respectively. All parameters were essentially within normal limits among female patients whereas the variable range of different parameters was recorded among male participants, therefore [p – value 0.20; insignificant]. Mean values and co-relation with comorbid illustrated in (Table-II & III respectively).

**Table-I:** Association of Clinical Manifestations with Biochemical Markers.

Diagnostic Test		Fever / Cough		p-value

		No	Yes	
Total Leukocyte Count	Raised	16(5.4%)	80(83.3%)	<.001*
	WNL	280(94.6%)	16(16.7%)	
Chest X-ray	Bilateral Patch	16(5.4%)	80(83.3%)	<.001*
	WNL	280(94.6%)	16(16.7%)	
Serum Ferritin	Raised	40(13.5%)	24(25%)	<.01*
	WNL	256(86.5%)	72(75%)	
Serum ALT	Raised	24(8.1%)	8(8.3%)	1.0
	WNL	272(91.9%)	88(91.7%)	
Serum Creatinine	Raised	-	24 (25%)	<.001*
	WNL	296(100%)	72(75%)	
Serum Sodium	Raised	-	16(16.7%)	<.001*
	WNL	296(100%)	80(83.3%)	
C – reactive Protein	Raised	24(8.1%)	24 (25%)	<.001*
	WNL	272(91.9%)	72(75%)	

* Significant p-value; p-value was calculated by applying Fischer Exact Test

**Table-II:** Mean Values of Biochemical Markers.

Diagnostic Test	n (%)	Range	Mean±Standard Deviation
Serum ALT	32(8.2%)	46.00 - 110.00	72.00±24.17
Serum Creatinine	24(6.1%)	127.00 - 170.00	145.66±18.39
Serum Sodium	16(4.1%)	147.00 - 151.00	149.00±2.06
C- Reactive Protein	48(12.2%)	7.00 - 10.00	8.50±.96

**Table-III:** Association of Comorbid with Biochemical Markers.

Diagnostic Test		HTN	DM	Asthma	
CXR	Bilateral Patch	8 (20%)	16 (66.7%)	-	<.001*
	WNL	32 (80.0%)	8 (33.3%)	8 (100%)	
Serum Creatinine	Raised	8 (20%)	8 (33.3%)	-	.14
	WNL	32 (80.0%)	16 (66.7%)	8 (100%)	
C- Reactive Protein	Raised	8 (20%)	16 (66.7%)	-	<.001*
	WNL	32 (80.0%)	8 (33.3%)	8 (100%)	
Total Leukocyte Count	Raised	8 (20%)	16 (66.7%)	-	<.001*
	WNL	32 (80.0%)	8 (33.3%)	8 (100%)	
Coagulation Profile	Deranged	8 (20%)	8 (33.3%)	-	.14
	WNL	32 (80.0%)	16 (66.7%)	8 (100%)	

* Significant p-value; p-value was calculated by applying Fischer Exact Test

## DISCUSSION

Study results has demonstrated that fever was the main presenting feature of Covid illness. No basic co-association gathered with sexual direction. Raised serum ferritin, C – reactive protein and chest x-ray discoveries call for multidisciplinary executives for disease management. Research examination collaborates with the various reported studies regarding the matter.

Fu et al completed a review preliminary in Suzhou China to investigate clinical ramifications of dynamic neutrophil to lymphocyte proportion and D-dimer in COVID-19 conceded patients. Evident variations from the norm of research facility blood assessments were found, particularly for peripheral blood cell evaluation and coagulation profile. Frequent findings were hemocytopenia, lymphopenia, leukopenia, and thrombocytopenia (45.3%, 21.3%, and 12% respectively). The pace of anaemia was (8%).The coagulation profile uncovered an undeniable rise of fibrinogen (60%) and D-dimer (12%) regardless of within normal limits prothrombin time (PT) and partial thromboplastin time (APTT). As to trial of the liver and renal function, tests were within stipulated range as in our study patients only serum ALT was deranged in (8.1%). The rise of the C-reactive protein level was seen in (58.7%) of coronavirus patients on the other hand our examination revealed (12.2%).^11^

Zhang et al dissected Clinical attributes of 140 patients contaminated with SARS-CoV-2 in Wuhan, China. Of the 135 patients with a chest CT scan on confirmation, the greater part (99.3%) had unusual outcomes, demonstrating run of the mill pictures that were reciprocal numerous ground-glass opacities or consolidation. The blood test consequence of patients upon the arrival of emergency clinic confirmation demonstrated normal leukocytes in the vast majority of the patients (68.1%), with 12.3% expanded and 19.6% diminished numbers. Lymphopenia was noticed in 75.4% of the patients. Most of these patients 52.9% had eosinopenia. Other research facility discoveries incorporated a higher convergence of C-reactive protein 91.9% and D-dimer 43.2%.^13^

Huang et al contemplated Clinical highlights of patients contaminated with 2019 novel coronavirus in Wuhan, China. The blood checks of patients on affirmation indicated leucopenia 25% patients and lymphopenia 63% patients. Coagulation Profile was deranged (p = 0.012) whereas in (16.3%) patients had deranged coagulation profile (p = 0.14). Levels of aspartate aminotransferase were expanded in 37% of 41 patients, including (62%) of 13 ICU patients and 25% of 28 non-ICU patients. Of the 41 patients, (98%) had respective various lobular and subsegmental zones of consolidation on CT Chest.^14^

Chen et al assessed employing distinct examination epidemiological and clinical qualities of 99 instances of 2019 novel coronavirus pneumonia in Wuhan, China. On confirmation, leucocytes were beneath the typical range in (9%) patients or more than normal range in (24%) patients. About 38% patients had neutrophils over the typical range. Lymphocytes (35%) and haemoglobin 51% were beneath the ordinary range in numerous patients Platelets were below the normal range in 12% patients or more than typical range in 4%. About 43% patients had contrasting degrees of liver capacity variation from the norm, with alanine aminotransferase (ALT) or aspartate aminotransferase (AST) over the stipulated range.Seven(7%) patients had varying degrees of renal function incapacitation with raised blood urea nitrogen or serum creatinine. Sixty three percent had serum ferritin above the typical range. 63 out of 73(86%) patients had elevated C-reactive protein.^15^

Xu et al inspected in a review study a Group of Patients Infected With Coronavirus Outside of Wuhan, China. On confirmation, the blood checks of 31% patients indicated leucopenia and 42% demonstrated lymphopenia contradictory to our trial participants where raised values noted in Total Leukocyte count among 24.5% patients. Levels of aspartate aminotransferase expanded in 16% patients. 84% patients demonstrated bilateral association on chest radiographs whereas figure was 24.5% study participants.^16^

Liu et al surveyed biochemical files from COVID-19 tainted patients. The most widely recognized research facility variations from the norm were hypoalbuminemia, lymphopenia, diminished level of lymphocytes and neutrophils, raised C-responsive protein, and lactate dehydrogenase. CRP was exceptionally associated with the intensity of lung injury. But co-relation in our study patients revealed to be insignificant (p-value = 0.15) as elaborated in (Table-IV) .^17^

**Table-IV:** Co-relation C-reactive Protein with Chest X-ray.

Diagnostic Test		CRP		p-value

		Raised	WNL
CXR	Bilateral Patch	16(33.3%)	80(23.3%)	.151*
	WNL	32(66.7%)	264(76.7%)

* Insignificant p-value; p-value was calculated by applying Fischer Exact Test

Wang et al examined the qualities of 34 children tainted with novel coronavirus Shenzhen. In the 34 cases, the white platelet checks of (82%) were typical. Five cases had expanded consistent with 24.5% infected patients of our examination and one case had diminished white cell checks.Neutropenia and lymphopenia was found in one case, individually. C-reactive protein levels and erythrocyte sedimentation rates were raised in 1 and 5 cases, separately. Raised D-Dimer found in 3 cases. The CT scan of these patients indicated bilateral sketchy or nodular ground-glass opacities.^18^

Chen et al made an investigation of 29 patients contaminated with coronavirus. The blood trial of the patients demonstrated ordinary or diminished white cell tally 23/29, diminished lymphocyte check 20/29, expanded C reactive protein 27/29. Albumin was decreased in 15/29, however, it was essentially within normal limits in 100% patients. Alanine aminotransferase (ALT), aspartate aminotransferase (AST), bilirubin, serum creatinine demonstrated no noteworthy changes.^19^

Chen et al assessed intrauterine vertical transmission capability of COVID-19 disease in nine pregnant patients. Information from research centre tests indicated that five of the nine parturients with COVID-19 pneumonia had lymphopenia.6 patients had raised C- reactive protein (>10 mg/L) relevant to our evaluation with the mean value of (8.50±.96). Three had elevated alanine aminotransferase (ALT) and aspartate aminotransferase (AST).^20^

### Limitations of the study

Our examinations were compelled to a restricted period and may have missed time-changing ailment highlights, given the quick advancement of the epidemic.It stays a provoking errand to contain a flare-up of a novel pathogen proficient for individual to individual transmission in this exceptionally portable world, specifically at the point when treatment and counteraction alternatives are constrained.

## CONCLUSION

Routine haematological tests, biochemical tests, serological tests, and radiographic tests are crucial to conclusion, foundation and progression of ailment in COVID-19 contaminated patients.

## RECOMMENDATIONS

Clinical preliminaries should be painstakingly planned and conceptualized to evaluate the disease process, which could be testing given the already overstretched medical resources. The continuous, exceptional episode of COVID-19 diseases comprehensively has underlined the significance of the research facility finding of human coronavirus contaminations to restrain the spread as well as properly treat those patients who have asymptomatic disease.

### Authors’ Contribution:

**SA:** Conceived, designed and did statistical analysis & editing of manuscript, is responsible for integrity of research.

**AR, BA & MS:** Did data collection and manuscript writing.
